# Autophagy activation mediates resistance to FLT3 inhibitors in acute myeloid leukemia with FLT3-ITD mutation

**DOI:** 10.1186/s12967-022-03498-1

**Published:** 2022-07-06

**Authors:** Dan Xu, Yishan Chen, Ying Yang, Zhao Yin, Changfen Huang, Qiang Wang, Ling Jiang, Xuejie Jiang, Changxin Yin, Qifa Liu, Guopan Yu

**Affiliations:** Department of Hematology, Nanfang Hospital, Southern Medical University, No. 1838 Guangzhou Avenue North, Guangzhou, 510515 China

**Keywords:** Autophagy, FLT3 inhibitor, Resistance, Acquired mutation, Bone marrow micro-environment, FLT3-ITD, Acute myeloid leukemia

## Abstract

**Background:**

Autophagy plays a critical role in drug resistance in acute myeloid leukemia (AML), including the subtype with FLT3-ITD mutation. Yet how autophagy is activated and mediates resistance to FLT3 inhibitors in FLT3-ITD-positive AML remains unsure.

**Methods:**

We detected the expression of autophagy markers in FLT3-ITD-positive leukemic cells after vs. before acquired resistance to FLT3 inhibitors; tested the stimulative effect of acquired D835Y mutation and bone marrow micro-environment (BME) on autophagy; explored the mechanism of autophagy mediating FLT3 inhibitor resistance.

**Results:**

Sorafenib-resistant cells markedly overpresented autophagy markers in comparison with sorafenib-sensitive cells or the cells before sorafenib treatment. Both acquired D835Y mutation and BME activated cytoprotective autophagy to mediate FLT3 inhibitor resistance. Autophagy activation decreased the suppression efficacy of FLT3 inhibitors on FLT3 downstream signaling and then weakened their anti-leukemia effect. Inhibition of autophagy with CQ significantly enhanced the suppressive effect of FLT3 inhibitor on FLT3 downstream signaling, in the end overcame resistance to FLT3 inhibitors.

**Conclusions:**

Autophagy might be stimulated by acquired mutation or BME, and bypass activate FLT3 downstream signaling to mediate FLT3 inhibitor resistance in FLT3-ITD-positive AML. Targeting autophagy could be a promising strategy to overcome resistance.

## Background

Fms-like tyrosine kinase 3-internal tandem duplication (FLT3-ITD) mutation is a common molecular event with an approximate incidence of 25% in acute myeloid leukaemia (AML) [1]. High allelic ratio (≥ 0.5) of FLT3-ITD is associated with a very poor prognosis in both adults and children, and are rarely cured by chemotherapy alone [2, 3]. Incorporation of FLT3 inhibitors with chemotherapy or haematopoietic stem cell transplantation has significantly improved the prognosis of FLT3-ITD-positive AML in recent years [4–6], but high incidence of leukaemia relapse remains a problem to be solved [2, 3]. Resistance to FLT3 inhibitors plays an important role in leukemia relapse [2, 7, 8]. The resistance mechanisms are known to mainly include overexpression of oncogenic kinases, FLT3 ligand overproduction, bone marrow micro-environment (BME)-mediated protection and acquired resistant mutation [2, 7].

Autophagy is an adaptive survival mechanism that is essential for cellular homeostasis in response to various stresses [9]. More and more studies [10–12] have linked alteration of autophagy with cancer initiation, progression and treatment resistance, including leukemia, thus autophagy has been shown to be a key therapeutic target. Most recently, Heydt et al. [13] reported that FLT3-ITD mutation increased basal autophagy to support leukemic cells survival; also autophagy inhibition overcame FLT3 inhibitor resistance in vitro and vivo, suggesting autophagy might involve in the development and progression of FLT3-ITD-positive AML. However, in this AML subtype, how autophagy is activated to induce resistance to FLT3 inhibitors, and how it mediates the resistance remains unclear. In this study, we mainly explored the correlation of autophagy with FLT3 inhibitor resistance, the inductive effect of acquired mutation and BME on autophagy, and the mechanism of autophagy mediating resistance.

## Materials and methods

### Reagents and antibodies

Sorafenib (CAS #475207-59-1) and quizartinib (AC220, CAS #950769-58-1) were purchased from Selleck. Chloroquince (CQ, CAS #50-63-5) was bought from Sigma. The antibodies against human-phosphorylated (p)-p44/42 MAPK (ERK1/2, Thr202/Tyr204, CAS #4370), p-FLT3 (Tyr589/591, CAS ##60413), p-mTOR (Ser2448, CAS #5536), p-S6K (Ser240/244, CAS #2215), mTOR (CAS ##2983), S6K (CAS #9202), Beclin-1 (CAS #4122), LC3B (CAS #3868), ATG5 (D5G3, CAS #9980), p62/SQSTM1 (CAS #5114), c-Myc (D84C12, CAS #5605) and cleaved caspase-3 (Asp175, CAS #9661) were purchased from Cell Signaling Technology. Against ERK2 (CAS #sc-1647) and FLT3 (CAS #sc-19635) were from Santa Cruz Biotechnology. Anti-GAPHD (CAS #G9545) was purchased from Millipore Sigma.

### AML patient samples and FLT3 mutant cell lines

Six patients with FLT3-ITD-positive AML were included from the ClinicalTrials (NCT02474290). The detail of clinical characteristics and treatment protocol had been reported [14]. Bone marrow samples were obtained from those patients at diagnosis, continued complete response (CCR) or relapsed after written informed consents were gotten according to the institutional guidelines of Medicine Institutional Review Boards of Nanfang Hospital, Southern Medical University. The mononuclear (MNC) cells in these samples were purified by Ficoll-Hypaque (Sigma-Aldrich) density gradient centrifugation, and then cultured in RPMI-1640 medium supplemented with 10% fetal calf serum (FCS).

Mesenchymal stem cells (MSCs), were obtained from bone marrows of one of the patients above at leukemia relapse (case #3) and one at the status of CCR (case #2), and cultured at a density of 5,000 cells/cm2 in a-MEM, supplemented with 20% FCS, 1% l-glutamine, and 1% penicillin–streptomycin. The MSCs were used for co-culture experiments after passage.

The Ba/F3-ITD, Ba/F3-D835Y and Ba/F3-ITD + D835Y cell lines, and the human AML cell line MOLM14 were all kindly provided by professor Andreeff Michael (Department of Leukemia Research, Division of Cancer Medicine, The University of Texas MD Anderson Cancer Center, Houston, TX) in 2016. All cell lines were validated by short tandem repeat (STR) DNA fingerprinting using the AmpFISTR Identifiler Kit as described before [15]. All cells were maintained in RPMI medium supplemented with 10% FCS.

### Cell viability and apoptosis assays

Cell viability was assessed using the trypan blue dye exclusion method, and apoptosis was determined via flow cytometry (FACS) by Annexin V positivity as described [16].

For measuring apoptosis induction in the leukemia cells co-cultured with MSCs, the cells were trypsinized and stained with CD90-PE, CD45-APC and Annexin V-FITC (all from BD Biosciences), and apoptosis was assessed by measuring Annexin V-FITC positivity after excluding the CD90 + CD45—(used as a MSC marker) cell population.

### Transmission electron microscopy

According to our previous report [17], samples were fixed with a solution containing 3% glutaraldehyde plus 2% paraformaldehyde in 0.1 M cacodylate buffer, pH 7.3, then washed in 0.1 M sodium cacodylate buffer and treated with 0.1% Millipore-filtered cacodylate buffered tannic acid, postfixed with 1% buffered osmium, and stained en bloc with 1% Millipore-filtered uranyl acetate. The samples were dehydrated in increasing concentrations of ethanol, infiltrated, and embedded in LX-112 medium. The samples were polymerized in a 60 ℃ oven for approximately 3 days. Ultrathin sections were cut in a Leica Ultracut microtome (Leica, Deerfield, IL), stained with uranyl acetate and lead citrate in a Leica EM Stainer, and examined in a JEM 1010 transmission electron microscope (JEOL, USA, Inc., Peabody, MA) at an accelerating voltage of 80 kV. Micrographs were taken at 7500 × or 50,000 × magnification.

### Immunoblotting analyses

The cells were treated with the indicated agents and then collected in lysis buffer. Phosphorylation and total protein levels were determined using Odyssey Infrared Imaging System (LI-COR Biosciences).

### Statistical analyses

The data are presented as the means ± SD of triplicate samples or assays. The statistical analyses were performed using unpaired Student t test. A 2-sided Fisher exact test was used to determine statistical significance between different groups. A P value ≤ 0.05 was considered statistically significant.

## Results

### FLT3 inhibitor resistant primary FLT3-ITD-positive AML cells showed overpresentation of autophagy markers

**Fig. 1:**
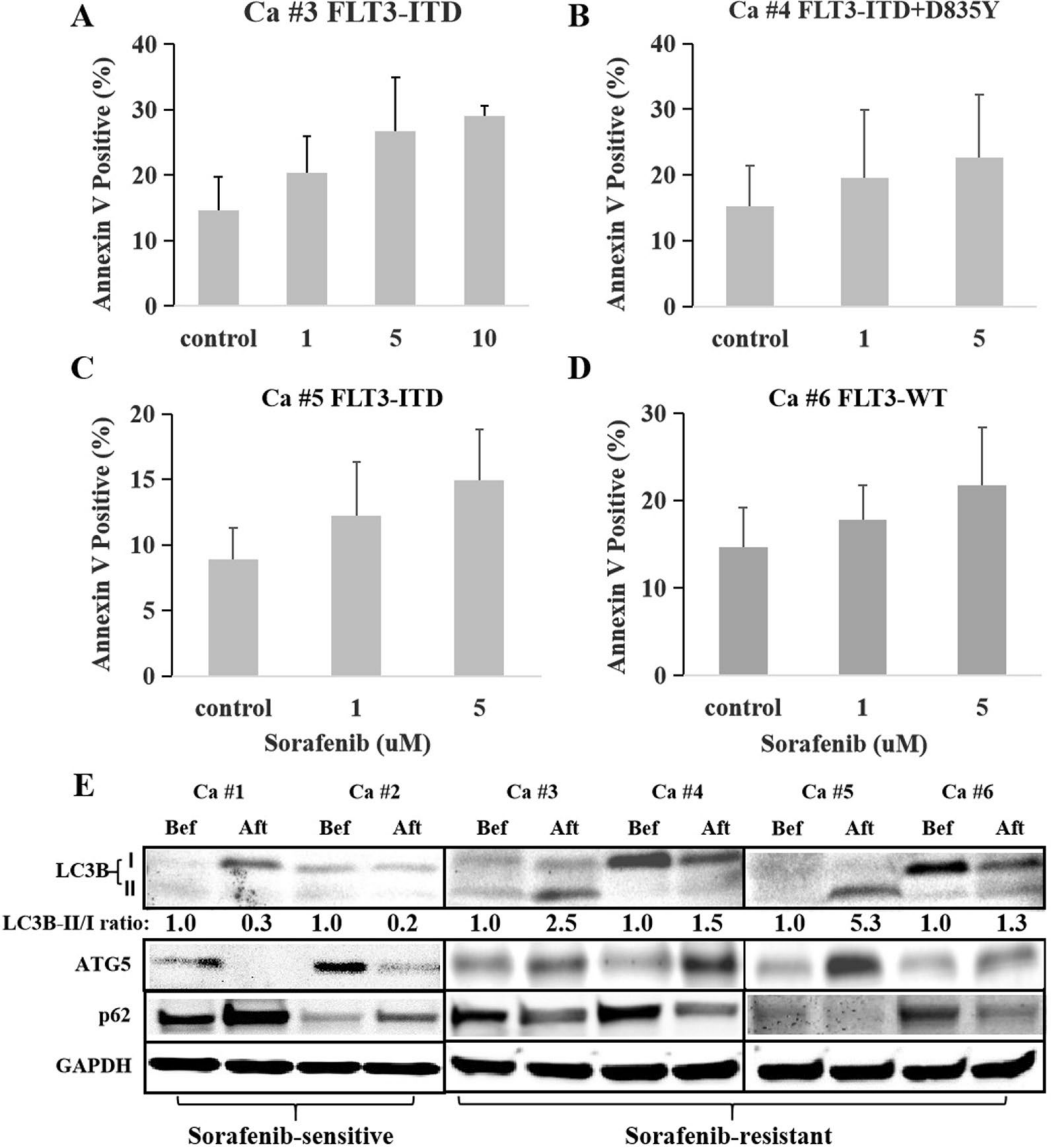
Sorafenib-resistant primary AML cells showed high expression of autophagy. **A**, **C** Primary AML cells with FLT3-ITD mutation from the patients (case #3 and #5) who relapsed on the maintenance sorafenib therapy were resistant to sorafenib. **B** Primary AML cells with FLT3-ITD + D835Y mutation from the patient (case #4) who relapsed on the maintenance sorafenib therapy were resistant to sorafenib. **D** Primary AML cells with FLT3-WT from the patient (case #6) who relapsed on the maintenance sorafenib therapy were resistant to sorafenib. **E** Sorafenib-sensitive primary AML cells from the patients with FLT3-ITD mutation who were continued complete response (CCR) during sorafenib therapy showed decreasing expression of LC3B-II after vs. before the treatment of sorafenib, showing as LC3B-II/I ratio lower than 1 (0.3 in case #1 and 0.2 in case #2). In contrast to the sorafenib-sensitive cells, sorafenib-resistant AML cells from the patients with FLT3-ITD mutation who relapsed on maintenance sorafenib therapy showed increasing expression of LC3B-II after relapse as compared with before the treatment, presenting as LC3B-II/I ratio higher than 1 (2.5 in case #3, 1.5 in case #4, 5.3 in case #5 and 1.3 in case #6). In accordance with the change of LC3B-II/I ratio, the expression of ATG5 decreased, and p62 accumulated in sorafenib-sensitive cells; while just being opposite, ATG5 increased, and p62 degraded in sorafenib-resistant cells after vs. before the treatment of sorafenib

Six patients with newly diagnosed FLT3-ITD-positiver AML, of whom 2 were CCR and 4 relapsed on sorafenib treatment, were included and isolated MNC cells from bone marrow at diagnosis and the status of CCR or relapse. The characteristics of the 6 patients were presented in Table 1. The primary AML cells, being isolated from 4 relapsed patients (Case #3 and #5 with FLT3-ITD mutation, case #4 with FLT3-ITD + D835Y mutation, and case #6 without FLT3 mutation at relapse) were truly resistant to sorafenib in vitro (Fig. 1A–D). In order to assess the expression of autophagy in sorafenib-resistant cells, we detected autophagy makers in those primary cells after vs. before sorafenib treatment. Immunoblotting analyses showed that, in comparison with the results before the treatment, LC3B-II/I ratio and ATG5 expression increased, and p62 degraded in the sorafenib-resistant blasts. In contrast, LC3B-II/I ratio and ATG5 expression decreased, and p62 accumulated in the sorafenib-sensitive cells (Fig. 1C), suggesting that FLT3 inhibitor resistant primary FLT3-ITD-positive AML cells might overexpress autophagy.

**Table 1:** Treatment response and FLT3 mutations of the 6 patients with FLT3-ITD-positive AML

Caseno.	Sex/age	Karyotype	Additional mutations	Sorafenib maintenance	Follow-up post allo-HSCT (M)	Response	FLT3 mutation			
							Diagnosis	AF	Relapse	AF
#1	M/52	46, XY	None	Yes	13.0	CCR	FLT3-ITD	0.62	NA	
#2	F/28	46, XY	TET2	Yes	8.0	CCR	FLT3-ITD	0.76	NA	
#3	M/39	47, XY, + 10	DNMT3A, NPM1, SOCS1	Yes	13.5	Relapse	FLT3-ITD	0.52	FLT3-ITD	0.39
#4	F/23	46, XX, t(11;20)(p15;q11)	TET2, RUNX1	Yes	10.0	Relapse	FLT3-ITD	0.43	FLT3-ITD + D835	0.30
#5	M/23	47, XY, + 8	TET2	Yes	11.5	Relapse	FLT3-ITD	0.66	FLT3-ITD	0.41
#6	M/43	46, XY	EZH2, RUNX1, BCL6	Yes	7.0	Relapse	FLT3-ITD	0.75	WT	

*Allo-HSCT* allogenetic hematopoietic stem cell transplantation; *M* months; *AF* allele frequency; *M* male; *CCR* continued complete response; *NA* not available; *F* female; *WT* wild type

### Acquired D835Y mutation induced resistance to FLT3 inhibitor and activated autophagy in FLT3-ITD-positive cell lines

**Fig. 2:**
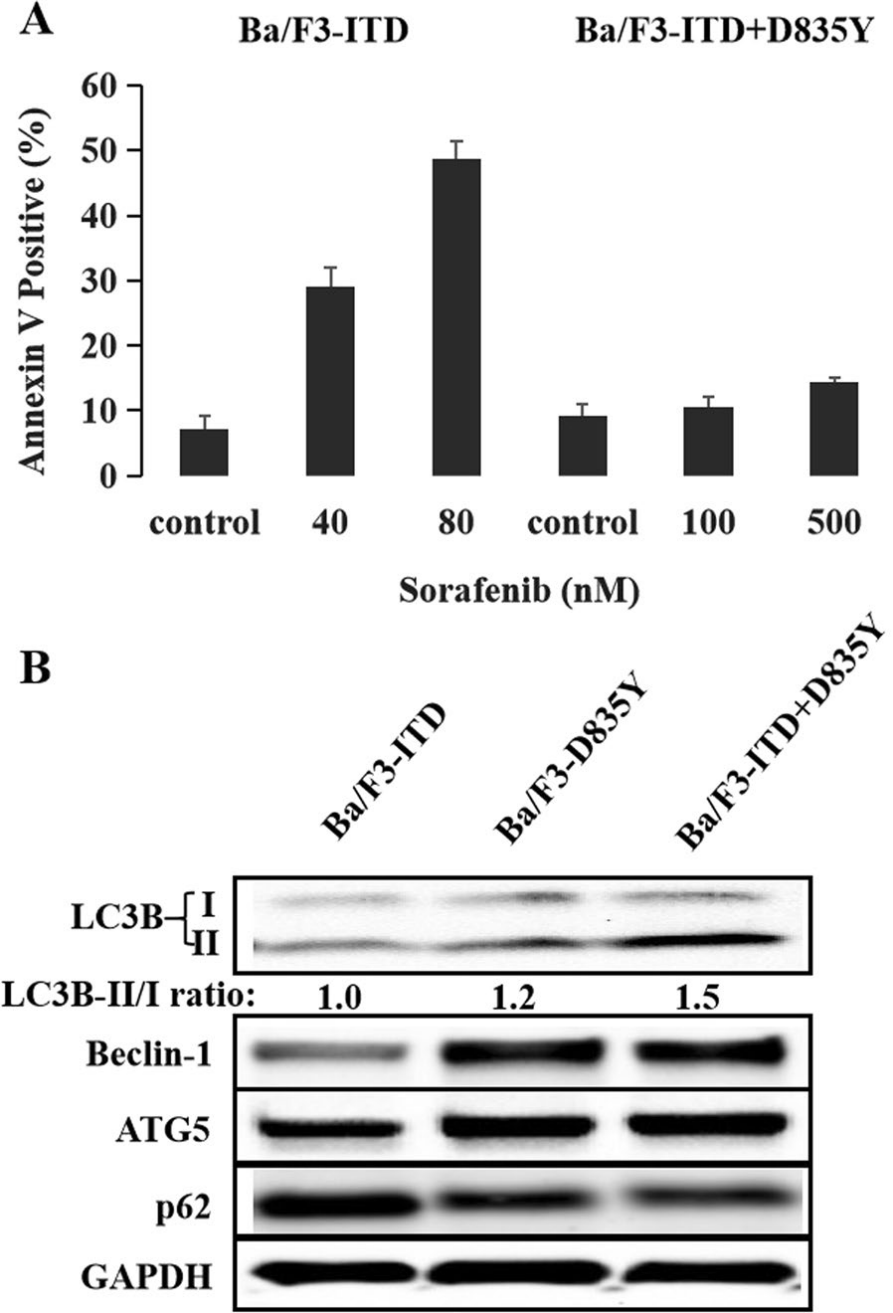
Sorafenib-resistant cell lines showed overpresentation of autophagy markers. **A** Baf/FLT3-ITD cell line was sensitive to sorafenib treatment with the apoptosis rate of 29.0 ± 3.1% at the concentration of 40 nM and 48.6 ± 2.9% at 80 nM. After acquiring D835Y mutation, Ba/F3-ITD + D835Y cell line showed resistant to sorafenib with the apoptosis rate of 10.5 ± 1.6% at the concentration of 100 nM and 14.3 ± 0.8% at 500 nM. **B** As being compared with Ba/F3-ITD cells, both Ba/F3-D835Y and Ba/F3-ITD + D835Y cells, which were resistant to sorafenib, showed increasing expression of LC3B-II (LC3B-II/I tatio: 1.0 in Ba/F3-ITD, 1.2 in Ba/F3-D835Y and 1.5 in Ba/F3-ITD + D835Y), Beclin-1 and ATG5, and decreasing p62

In order to test activation of autophagy in sorafenib-resistant AML cells, sorafenib-resistant cell lines were built such as Baf3 cells with FLT3-D835Y or FLT3-ITD + D835Y mutation and detected the expression of autophagy markers including LC3B, Beclin-1, ATG5 and p62. In line with previous report [18], Ba/F3-ITD + D835Y mutant cells were resistant to sorafenib (Fig. 2A). Compared with Ba/F3-ITD mutant cells, which was sensitive to sorafenib (Fig. 2A), both Ba/F3-D835Y and Ba/F3-ITD + D835Y mutant cells presented higher expression of LC3B-II, Beclin-1 and ATG5, and degratation of p62 (Fig. 2B), indicating that sorafenib-resistant FLT3-ITD-positive cell lines could also overexpress autophagy and the cytoprotective autophagy might be activated by acquired resistant mutation..

### Inhibition of autophagy overcame FLT3 inhibitor resistance in FLT3-ITD-positive AML

**Fig. 3:**
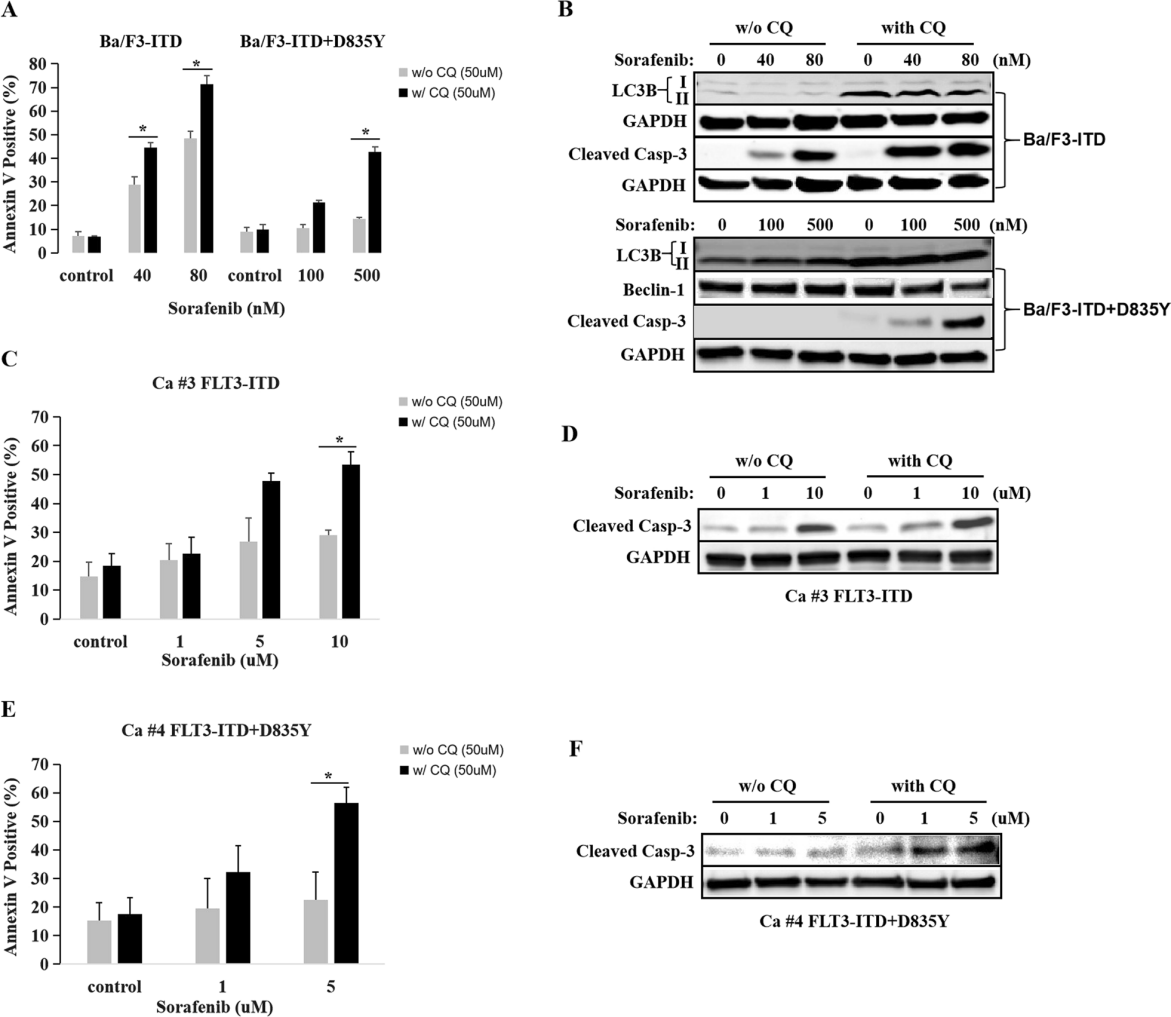
Inhibition of autophagy enhanced the anti-leukemia effect of sorafenib in FLT3-mutated leukemia cells. **A** Inhbition of autophagy with CQ, the anti-leukemia effect of sorafenib in both Ba/F3-ITD (w/o CQ vs. w/CQ: 40 nM, 29.0 ± 3.1% vs. 44.5 ± 2.2%, P = 0.038; 80 nM, 48.6 ± 2.9% vs. 71.5 ± 3.5%, P = 0.037) and Ba/F3-ITD + D835Y cells (w/o CQ vs. w/CQ, 500 nM, 14.3 ± 0.8% vs. 42.8 ± 2.1%, P = 0.006) was enhanced. **B** Western blot showed sorafenib significantly increased the expression of cleaved-caspase 3 in both Ba/F3-ITD and Ba/F3-ITD + D835Y cells after being dealt with vs. without CQ. **C** CQ enhanced the anti-leukemia effect of sorafenib in sorafenib-resistant primary AML cells with FLT3-ITD mutation (w/o CQ vs. w/CQ, sorafenib at 5 µM, 26.7 ± 8.2% vs. 47.8 ± 2.7%, P = 0.075; sorafenib at 10 µM, 29.0 ± 1.6% vs. 53.5 ± 4.3%, P = 0.018). **D** Western blot showed sorafenib significantly increased the expression of cleaved-caspase 3 in sorafenib-resistant primary AML cells with FLT3-ITD mutation (Ca #3) after being dealt with vs. without CQ. **E** CQ enhanced the anti-leukemia effect of sorafenib in sorafenib-resistant primary AML cells with FLT3-ITD + D835Y mutation (w/o CQ vs. w/CQ, sorafenib at 5uM, 22.6 ± 9.6% vs. 56.5 ± 5.4%, P = 0.049). **F** Western blot showed sorafenib significantly increased the expression of cleaved-caspase 3 in sorafenib-resistant primary AML cells with FLT3-ITD + D835Y mutation (Ca #4) after being dealt with vs. without CQ

The data above showed that sorafenib-resistant leukemia cells expressed higher autophagy, suggesting autophagy activation might be associated with FLT3 inhibitor resistance in FLT3-ITD-positive AML. To test this hypothesis, we used CQ to down-regulate autophagy in sorafenib-resistant cells, then detected whether it would strengthen the anti-leukemia effect of sorafenib. As mention above, Ba/F3-ITD + D835Y cells were resistant to sorafenib. After inhibition of autophagy with CQ, Ba/F3-ITD + D835Y cells were turned to be sensitive to sorafenib treatment (Fig. 3A). In line with this, western blot showed sorafenib increased the expression of cleaved-caspase 3 in Ba/F3-ITD + D835Y cells after being dealt with vs. without CQ (Fig. 3B). In addition, inhibition of autophagy with CQ also enhanced the anti-leukemia effect of sorafenib in Ba/F3-ITD cells (Fig. 3A and B). Furthermore, sorafenib-resistant primary AML cells with FLT3-ITD mutation (case #3, Fig. 3C and D) or FLT3-ITD + D835Y mutation (case #4, Fig. 3E and F) were also sensitized to sorafenib after being dealt with CQ. Taking together, sorafenib-resistant leukemia cells overpresented autophagy markers; inhibition of autophagy partly overcame sorafenib resistance, suggesting activation of autophagy could be an important factor for FLT3 inhibitor resistance in FLT3-ITD-poisitive AML cells.

### Inhibition of autophagy enhanced the suppression efficacy of FLT3 inhibitor on FLT3 downstream signaling

**Fig. 4:**
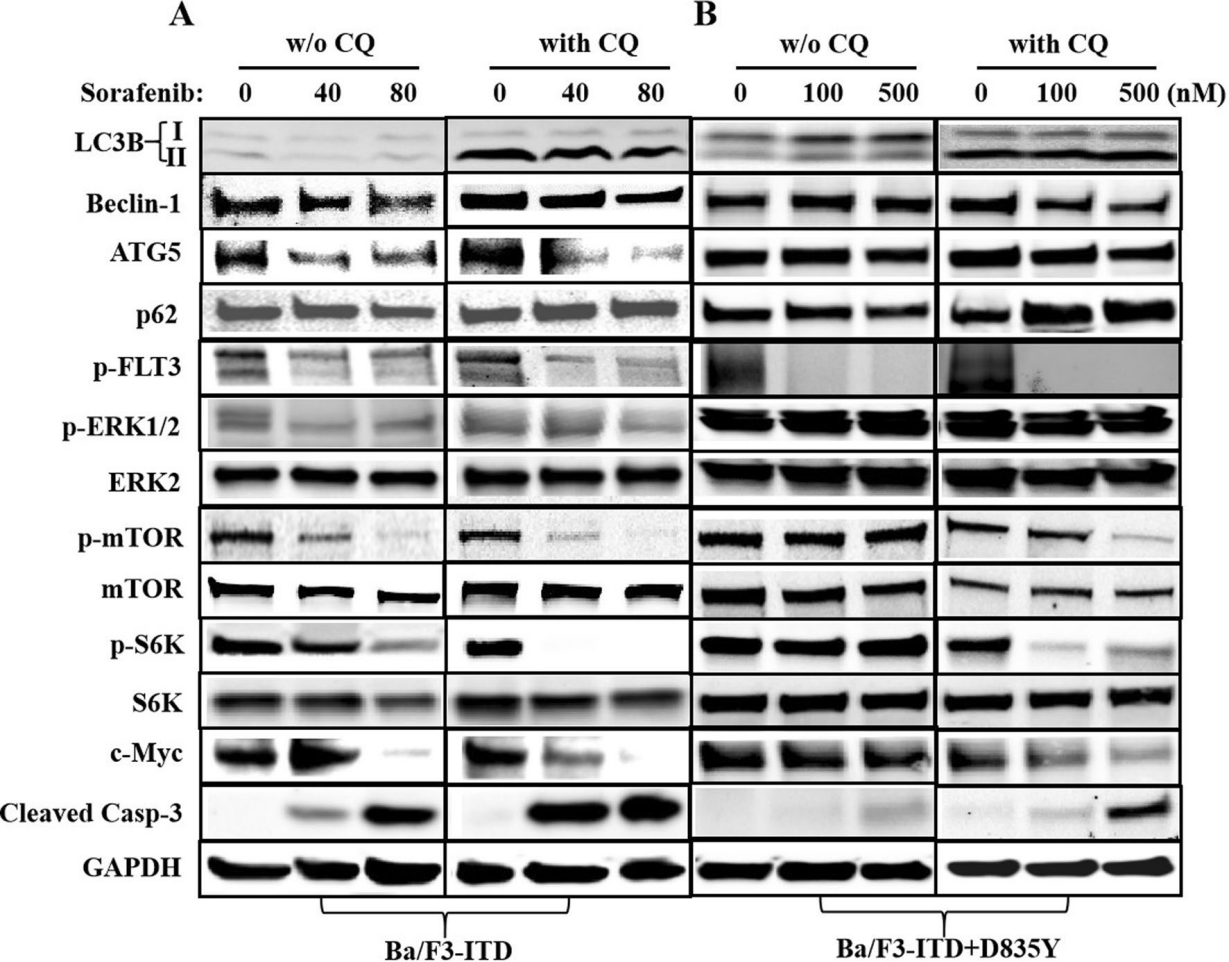
Inhibition of autophagy enhanced suppression of sorafenib on FLT3 downstream signaling and pro-apoptotic effect in both Ba/F3-ITD and Ba/F3-ITD + D835 mutated cells. **A** In Ba/F3-ITD cells, after autophagy was inhibited with CQ, sorafenib more markedly suppressed FLT3 downstream signaling of significantly induced cleaved caspase-3. **B** In Ba/F3-ITD + D835Y cells, without CQ co-treatment, sorafenib could only down-regulate the expression of p-FLT3, but not FLT3 downstream signaling, and no apparent cleaved caspase-3 was induced. After being dealt with CQ, autophagy was inhibited, sorafenib significantly suppressed the downstream signaling of FLT3 and up-regulated the expression of cleaved caspase-3

We then went further to explore how sorafenib worked after autophagy was inhibited. Immunoblotting data showed that, in Ba/F3-ITD + D835Y cells, without CQ co-treatment, sorafenib could only obviously down-regulate the expression of p-FLT3, but not FLT3 downstream signaling, and no apparent pro-apoptotic affect was induced. However, after being dealt with CQ, autophagy was inhibited, sorafenib significantly suppressed the downstream signaling of FLT3 and markedly induced the expression of cleaved caspase-3 (Fig. 4B), which accorded with autophagy inhibition enhancing the killing effect of sorafenib in Ba/F3-ITD + D835Y cells. In accordance with that, in sorafenib-sensitive cell line Ba/F3-ITD, after autophagy was inhibited with CQ, FLT3 downstream signaling was more markedly suppressed and cleaved caspase-3 was significantly induced by sorafenib (Fig. 4A). All these data indicated that autophagy might bypass activate FLT3 downstream signaling to decrease the cytotoxic effect of FLT3 inhibitor in FLT3 inhibitor resistant even sensitive leukemic cells.

### Bone marrow micro-environment mediated FLT3 inhibitor resistance in FLT3-ITD mutant cells via activating autophagy

It is reported that BME could up-regulate autophagy to mediate chemotherapy resistance in AML [19]. Whether BME-mediated autophagy could induce FLT3 inhibitor resistance in FLT3-ITD-positive AML remains unsure. To test the affect of BME on activation of autophagy and mediation of FLT3 inhibitor resistance in FLT3-ITD mutant cells, we detected the expression of autophagy markers and the anti-leukemia effect of FLT3 inhibitors including sorafenib and AC220 in FLT3-ITD-mutated cells with vs. without MSCs (case #3) co-culture.

**Fig. 5:**
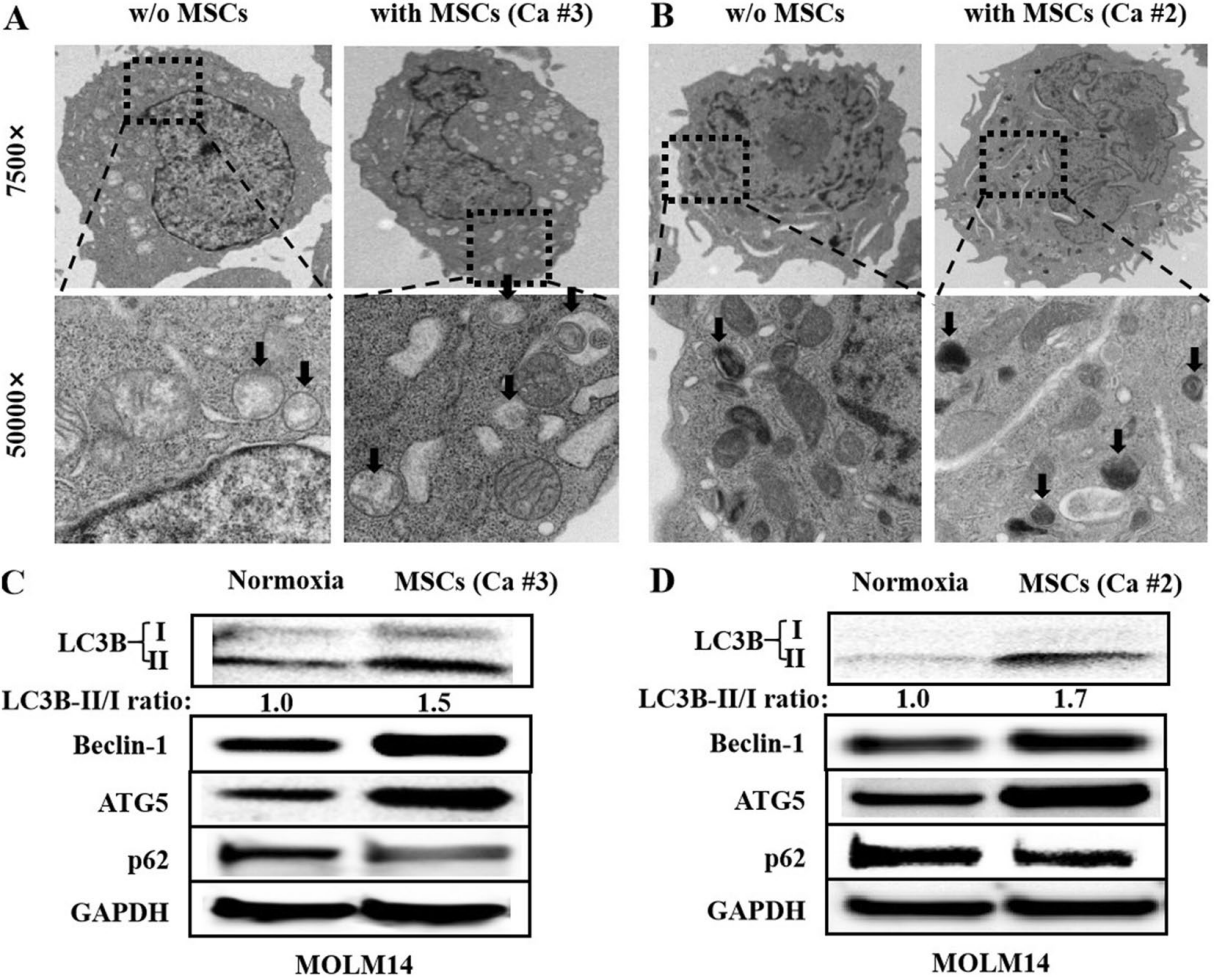
Co-culture with MSCs up-regulated autophagy markers in AML cells with FLT3-ITD mutation. As compared with that without MSCs co-culture, MSCs, no matter from the relapsed patient (case #3, **A**) or from the CCR case (case #2, **B**) with FLT3-ITD mutation, significantly increased the number of phagosomes (under the arrows) in MOLM14 cells being detected with transmission electron microscopy. Immunoblotting analyse showed MSCs, from the relapsed patient (case #3, **C**) or from the CCR case (case #2, **D**) with FLT3-ITD mutation, increased the expression of LC3B-II, Beclin-1 and ATG5, and degraded p62 expression in MOLM14 cells

**Fig. 6:**
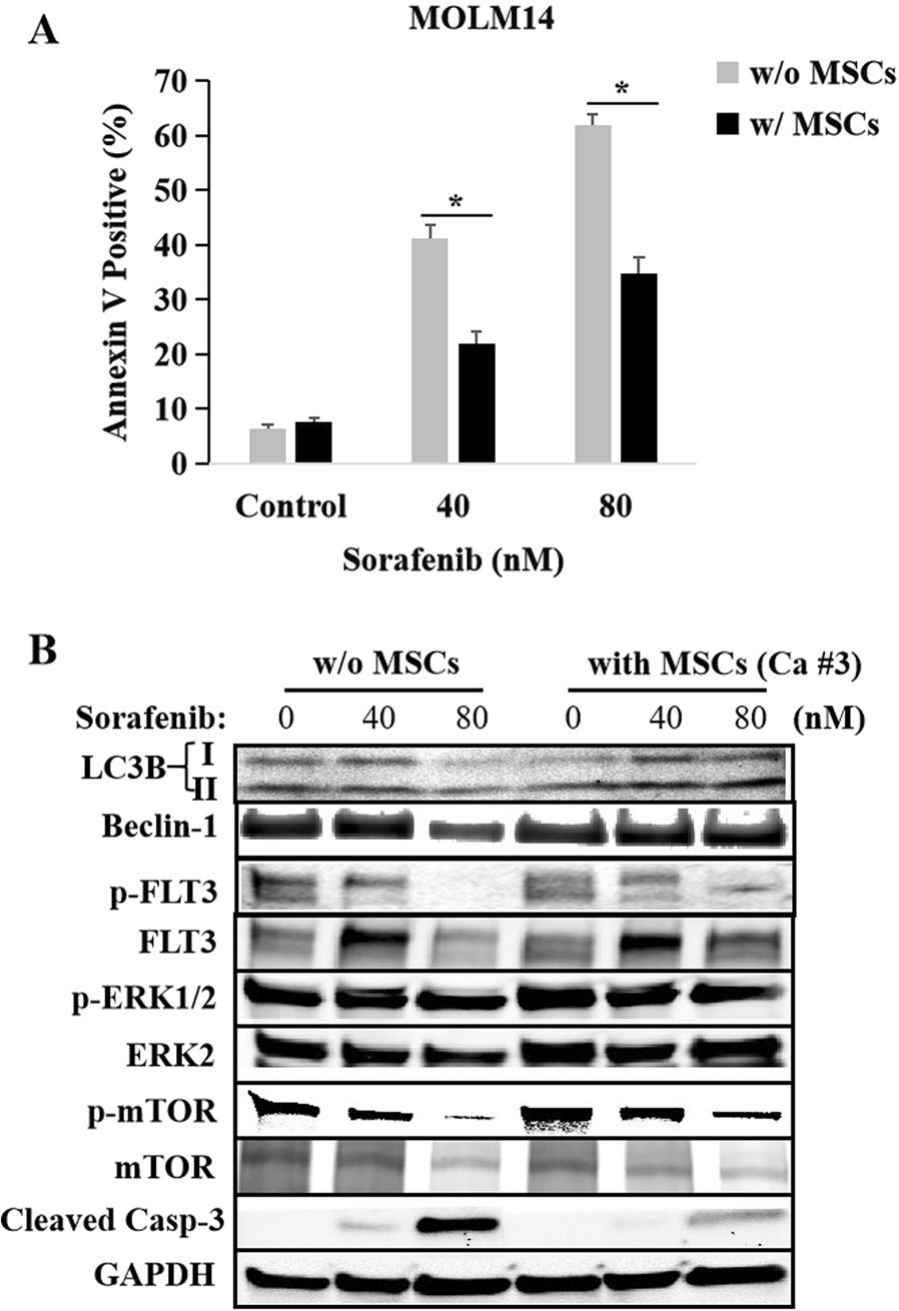
MSCs decreased the anti-leukemia effect of sorafenib via upregulation of autophagy in FLT3-ITD-positive AML cells. **A** After co-culture with MSCs from case #3, the killing effect of sorafenib in MOLM14 cells decreased significantly, showing as the apoptosis rate of 22.0% ± 2.1% vs. 41.2% ± 2.5% without MSCs (P = 0.014) at 40 nM and 34.8% ± 3.0% vs. 61.9% ± 1.9% (P = 0.008) at 80 nM, respectively. **B** Immunoblotting analyse showed that, as compared with that without MSCs co-culture, MSCs from case #3 up-regulated the expression of LC3B-II and Beclin-1 and remarkably decreased the inhibition efficacy of sorafenib on FLT3 pathway, especially on FLT3 downstream signaling, including p-FLT3, p-ERK1/2 and p-mTOR, and then decreased the induction of cleaved caspase 3 in MOLM14 cells

**Fig. 7:**
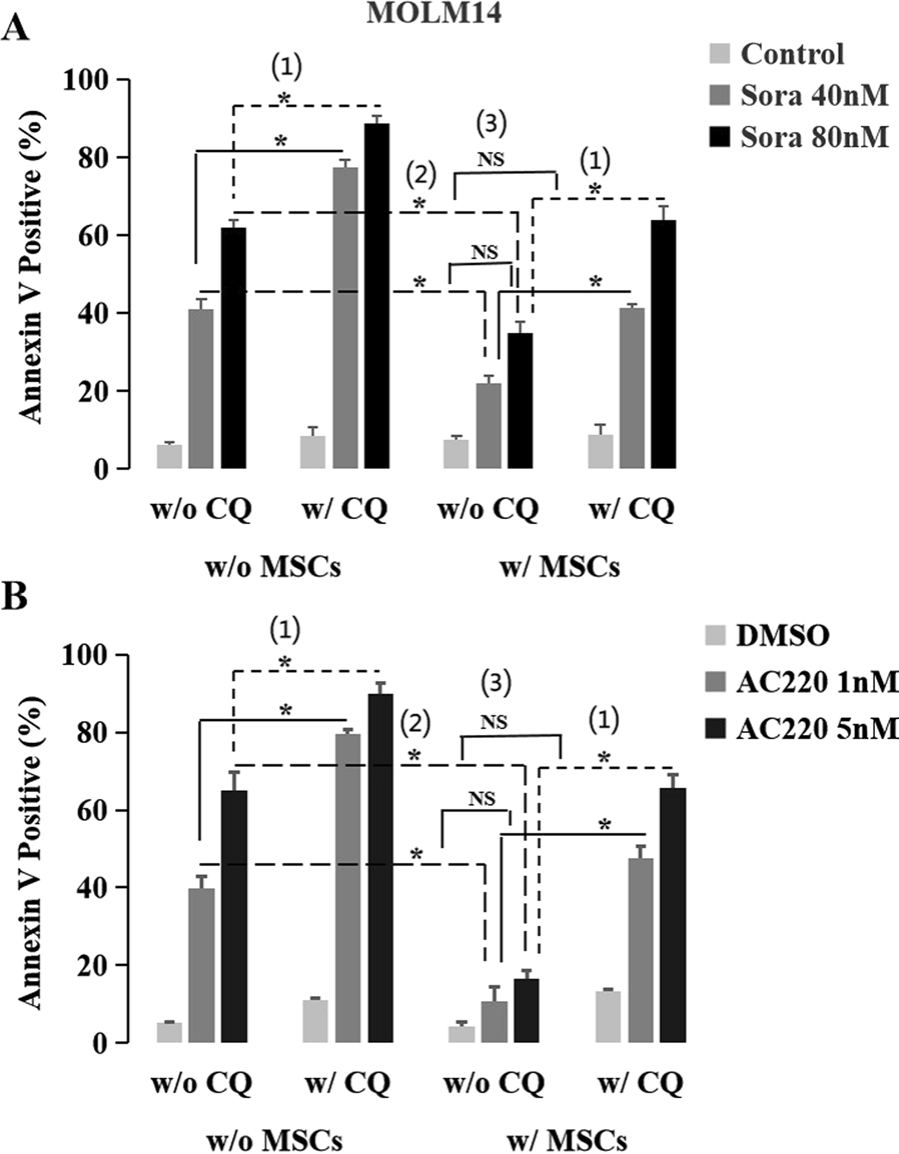
Inhibition of autophagy enhanced the anti-leukemia effect of FLT3 inhibitors and overcame MSCs-mediated resistance in FLT3-ITD-positive AML cells. **A1** Inhibition of autophagy enhanced the anti-leukemia effect of sorafenib. Regardless of MSCs co-culture or not, CQ significantly enhanced the killing effect of sorafenib in MOLM14 cells, showing as: without MSCs co-culture, the apoptosis rate of 41.2% ± 2.5% w/o CQ vs. 77.4% ± 2.1% w/CQ (P = 0.004) at the concentration of 40 nM, 62.0% ± 1.9% vs. 88.7% ± 2.1% (P = 0.005) at 80 nM; With MSCs co-culture, 22.0% ± 2.1% w/o CQ vs. 41.2% ± 1.3% w/CQ (P = 0.008) at 40 nM, 34.8% ± 3.0% vs. 63.9% ± 3.7% (P = 0.013) at 80 nM, respectively. **A2** MSCs decreased the anti-leukemia effect of sorafenib. With MSCs co-culture, the killing effect of sorafenib in MOLM14 cells decreased significantly, showing as the apoptosis rate of 22.0% ± 2.1% vs. 41.2% ± 2.5% w/o MSCs (P = 0.014) at 40 nM and 34.8% ± 3.0% vs. 61.9% ± 1.9% (P = 0.008) at 80 nM, respectively. **A3** Inhibition of autophagy overcame MSCs-mediated sorafenib resistance. Though co-culture with MSCs, after being dealt with CQ, the killing effect of sorafenib was similar to that w/o MSCs co-culture, showing as the apoptosis rate of 41.2% ± 1.3% vs. 41.2% ± 2.5% w/o CQ w/o MSCs (P > 0.05) at 40 nM and 63.9% ± 3.7% vs. 62.0% ± 1.9% w/o CQ w/o MSCs (P > 0.05).at 80 nM, respectively. **B1** Inhibition of autophagy enhanced the anti-leukemia effect of AC220. **B2** MSCs decreased the anti-leukemia effect of AC220. **B3** Inhibition of autophagy overcame MSCs-mediated AC220 resistance

After co-culture with MSCs, no matter from the relapsed patient (case #3) or from the CCR case (case #2) with FLT3-ITD mutation, the number of phagosomes in MOLM14 cells being detected with transmission electron microscopy showed significant increase (Fig. 5A and B). In accordance with this, the expression of LC3B-II, Beclin-1 and ATG5 up-regulated, and p62 degraded with vs. without MSCs co-culture, with western blot assessment (Fig. 5C and D). In addition, MSCs from case #3 significantly decreased the killing effect of sorafenib either at the concentration of 40 nM or 80 nM in MOLM14 cells (Fig. 6A). MSCs-mediated protection on MOLM14 cells was also found in the treatment of AC220 (Fig. 7B2). In agreement with this, the immunoblotting data showed that in contrast to the result without MSCs co-culture, MSCs from case #3 upregulated the expression of LC3B-II and Beclin-1 in MOLM14 cells and weakened the inhibition efficacy of sorafenib on FLT3 signaling pathway, especially on FLT3 downstream signaling, presenting as reducing suppression on p-FLT3, p-ERK1/2 and p-mTOR with MSCs co-culture, and then decreased the expression of cleaved-caspase 3 (Fig. 6B), which could explain MSCs decreased the anti-leukemia effect of sorafenib or AC220 in MOLM14 cells, suggesting that MSCs-mediated FLT3 inhibitor resistance in FLT3-ITD mutant cells might be associated with activating autophagy.

Then we used CQ to inhibit autophagy in MOLM14 cells. After inhibition of autophagy, MSCs-mediated resistance to sorafenib (Fig. 7A2) or AC220 (Fig. 7B2) was overcome (Fig. 7A3 and B3), and even the anti-leukemia effect of sorafenib (Fig. 7A1) or AC220 (Fig. 7B1) was sensitized regardless of MSCs co-culture, in agreement with the report of FLT3-ITD up-regulating autophagy to mediate resistance to FLT3 inhibitors in AML [13]. Taking together, BME-mediated FLT3 inhibitor resistance might be associated with MSCs-inducing autophagy; Inhibition of autophagy could partly overcome BME-mediated resistance to FLT3 inhibitors, in FLT3-ITD-positive AML.

## Discussion

FLT3 inhibitor resistance is the important reason for leukemia relapse in FLT3-ITD-positive AML [2, 7, 8]. In the present study, we revealed that, in FLT3-ITD-positive AML, FLT3 inhibitor resistant cells overexpressed autophagy; Autophagy was activated by acquired D835Y mutation or BME and then mediated FLT3 inhibitor resistance; Autophagy activation reduced the suppression efficacy of FLT3 inhibitors on FLT3 downstream signaling and then decreased their pro-apoptotic effect; Inhibition of autophagy enhanced the anti-leukemia effect of FLT3 inhibitors, partly overcame FLT3 inhibitor resistance,. Our data further supports that autophagy significantly involves in leukemia progression and resistance, could be a promising therapeutic target in FLT3-ITD-positive AML.

Our results accord with previous studies [10–13, 20, 21], that autophagy is closely associated with resistance in AML, especially in the patients with FLT3-ITD mutation. Activation of cytoprotective autophagy is found in cytarabine-resistant AML cells, and blockade of autophagy markedly increases the cytotoxic effect of cytarabine [21, 22]. Oncogenic FLT3-ITD increases autophagic flux to support AML cell survival and proliferation. Inhibition of autophagy overcomes FLT3 inhibitor resistance in FLT3-ITD-positive cells [13, 23]. In our study, FLT3 inhibitor resistant leukemia cells showed significantly activating autophagy. When autophagy was inhibited, those resistant cells were sensitized to FLT3 inhibitors.

In this study, acquired D835Y mutation and BME were found to be important factors for stimulation of autophagy in FLT3-ITD-positive cells. As it is known that acquired mutation is the key factor for resistance to FLT3 inhibitors in FLT3-ITD-positive AML [2, 7, 8]. Except for changing molecular conformation [24], we revealed that, autophagy activation by acquired D835Y mutation could be an important mechanism for resistance. Inhibition of autophagy enhanced the anti-leukemia effect of sorafenib in leukemia cell lines and primary blasts with FLT3-ITD + D835Y mutation, which opens a window for overcoming FLT3 inhibitor resistance in AML with acquired D835 mutation. In addition, secondary mutation is acquired under clone selective pressure [25]. Since autophagy is an adaptive survival mechanism for leukemia cells in response to various stresses [9, 10, 12], the causal link between acquired mutation and autophagy activation remains open. In line with stimulation of cytoprotective autophagy against cytarabine/anthracycline combination by BME [19], our study also observed BME induced cytoprotective autophagy against FLT3 inhibitors. The way of BME stimulating autophagy needs further research.

Many studies [26–28] show that autophagy selectively eliminates impaired or extra intracellular contents, and then supports the maintenance and self-renewal capacity of cancer stem cells, acting as a regulatory or cytoprotective adaptive mechanism, leading to malignant progression of different types of cancers including AML. In FLT3-ITD-positive AML, FLT3-ITD mutation increases basal autophagy to support leukemic cell survival and proliferation via transcription factor ATF4 (activating transcription factor 4). Inhibition of autophagy or ATF4 enhances the anti-leukemia effect of FLT3 inhibitors [13]. Yet, how autophagy mediates FLT3 inhibitor resistance remains unclear. In our study, we found that the suppression of sorafenib on FLT3 downstream signaling and the induction of pro-apoptotic effect in FLT3-ITD-positive cells were weaken by autophagy activated by acquired D835Y mutation or MSCs. After autophagy was inhibited, sorafenib more markedly suppressed FLT3 downstream signaling and promoted cell death, and finally overcame FLT3 inhibitor resistance mediated by acquired mutation or MSCs, suggesting that autophagy overexpression might bypass activate FLT3 downstream signaling to eliminate the suppressive effect of FLT3 inhibitors on FLT3 pathway, which calls for further study.

More and more studies have demonstrated autophagy could be a promising therapeutic target for overcoming drug resistance in AML [11–13, 19, 20]. As a survival mechanism to resist cytotoxic stress, leukemia cells are found to increase autophagy during the treatment of chemotherapy including cytarabine and daunorubicin [12, 19, 29], or targeted therapy such as BET inhibitors [20], histone methyltransferase inhibitors [30], BCL2 inhibitors [31], to counteract therapeutic effect. Inhibition of autophagy could sensitize leukemia cells to these agents. In the present study, autophagy was also observed to be activated and mediated resistance to FLT3 inhibitors during the treatment in FLT3-ITD-positive AML. Targeted suppression of autophagy with CQ enhanced the anti-leukemia effect of FLT3 inhibitors, and eliminated acquired resistance mediated by acquired D835Y mutation or MSCs. In agreement with our data, Qiu et al. reported combination of quizartinib with autophagy inhibitor Lys05 markedly improved proliferation inhibition and apoptosis induction in comparison with quizartinib alone [23]. Heydt et al. showed autophagy suppression by SAR405 or shRNA against ATG12 overcame quizartinib resistance in MOLM-14 cells with FLT3-D835Y mutation in vitro and vivo [13]. All of these further support autophagy should be a critical target in AML therapy, especially in overcoming drug resistance.

## Conclusion

Our findings demonstrate autophagy activation is closely associated with FLT3 inhibitor resistance in FLT3-ITD-positive AML. Autophagy might be stimulated by acquired mutation or BME, and then decrease the anti-leukemia effect of FLT3 inhibitors via bypass activation of FLT3 downstream signaling. Targeting autophagy could be a promising strategy to overcome FLT3 inhibitor resistance.

## Data Availability

All data generated or analyzed during this study are included in this manuscript.

## Abbreviations

FLT3-ITD: Fms-like tyrosine kinase 3-internal tandem duplication
AML: Acute myeloid leukaemia
BME: Bone marrow micro-environment
CQ: Chloroquince
CCR: Continued complete response
MNC: Mononuclear
FCS: Fetal calf serum
MSCs: Mesenchymal stem cells
STR: Short tandem repeat
FACS: Flow cytometry
SD: Standard deviation
BET: Bromodomain and extra-terminal
BCL2: B-cell lymphoma 2
ATG5: Autophagy-related gene 5
ATG12: Autophagy-related gene 12
